# A Novel Treatment for Skin Manifestations in Dermatomyositis: A Case Report

**DOI:** 10.7759/cureus.66704

**Published:** 2024-08-12

**Authors:** Manasa Srinivasa Murthy, Ammar Haikal

**Affiliations:** 1 Internal Medicine, Bayonne Medical Center, Bayonne, USA; 2 Rheumatology, Hackensack University Medical Center, Hackensack, USA

**Keywords:** concomitant cutaneous hemangioma, photosensitive rash, novel therapy, interferon-mediated disease, interferon, anifrolumab, chronic pruritis, pruritis, s: dermatomyositis, dermatomyositis

## Abstract

We present a case of refractory cutaneous dermatomyositis (DM) in a 51-year-old Hispanic female which failed multiple treatments but found symptomatic relief with anifrolumab. Anifrolumab was the only treatment that was associated with significant improvement in the rash and pruritis of the patient and lowered her corticosteroid needs. To our knowledge, this is the only second case report that has shown success in treating refractory cutaneous symptoms of DM with anifrolumab after failing standard and multiple combinations of therapies.

Anifrolumab is a new first-in-class human monoclonal antibody, which inhibits type 1 interferon receptor (IFN-1) and is used to treat systemic lupus erythematosus (SLE). It is FDA-approved for non-renal manifestations of SLE. This IFN pathway seems to be also active in patients with DM. The presence of IFN-1 and IFN-2 has been reported in muscle biopsies of patients with inflammatory myopathies. Moreover, the IFN activation signature is present in the muscle, blood, and skin of patients with DM. IFN-1 has been assumed to activate toll-like receptors which activate the dendritic cells leading to the secretion of cytokines and chemokines. This potential pathophysiological role of IFN in DM may explain the symptom improvement experienced by our patient after starting anifrolumab treatment. Anifrolumab has additionally been shown to have a good safety profile when used to treat patients with SLE with up to three years of treatment on background conventional disease-modifying antirheumatic drug (DMARD) therapies.

In conclusion, SLE and DM share similarities in their pathophysiology and cutaneous disease involvement and can be differentiated clinically. Skin manifestations of DM can persist despite combinations of therapies even when weakness resolves. With this case report, we aim to highlight the possibility of utilizing anifrolumab for treating DM skin manifestations, especially in refractory cases. More research is needed to guide where anifrolumab stands in the therapeutic algorithm for DM. It is unknown whether it treats the myositis component, DM-related arthritis, or coexistent rheumatoid arthritis.

## Introduction

Dermatomyositis (DM) is an inflammatory myopathy associated with cutaneous manifestations such as shawl and V sign rash, heliotrope rash [1], and the pathognomonic Gottron papules [2]. Chronic pruritis, telangiectasias, calcinosis, and panniculitis are some atypical skin manifestations of DM [3]. Treatment strategies for skin manifestations include corticosteroids and topical calcineurin inhibitors, photoprotection for mild cases [4]. In moderate to severe disease, methotrexate (MTX), hydroxychloroquine (HCQ), and mycophenolate mofetil (MMF) are used [5]. We present the case of a 51-year-old female who failed several treatments for refractory painful and pruritic DM rash and finally found relief with anifrolumab.

## Case presentation

**Figure 1:**
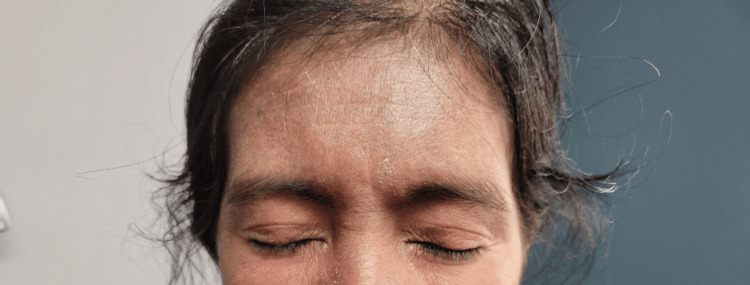
Purplish/violaceous erythema over the eyelids causing itching and photosensitivity (heliotrope rash)

**Figure 2:**
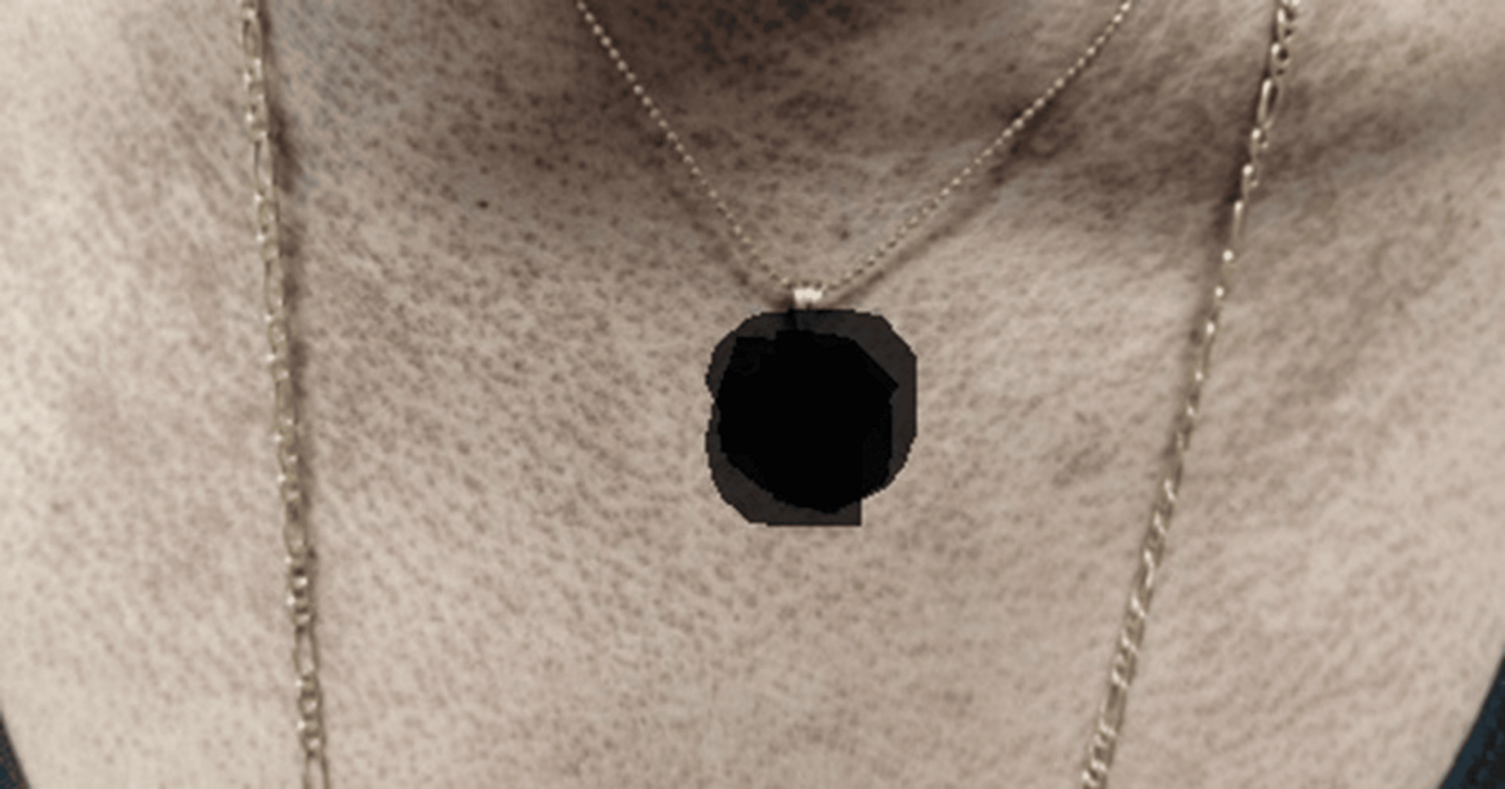
Pruritic painful rash on the anterior chest

**Figure 3:**
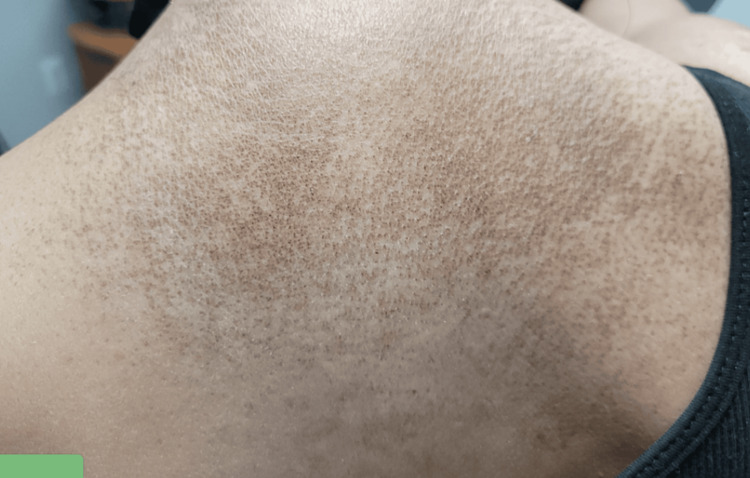
Painful pruritic rash on the upper back

**Figure 4:**
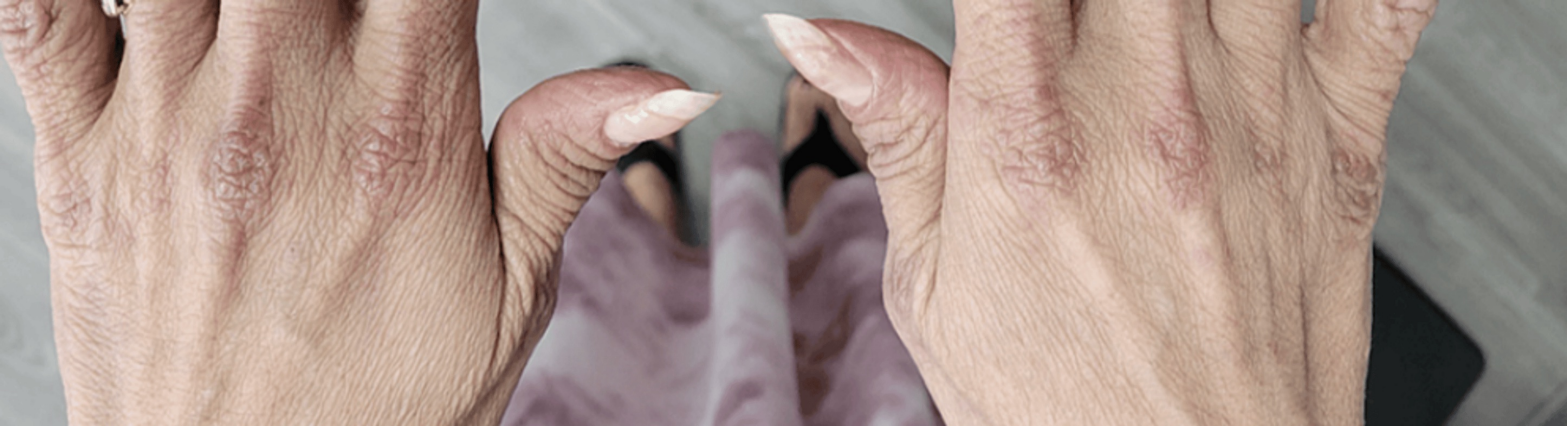
Scaly, itchy violaceous papules over the bilateral knuckles (Gottron's papules)

**Figure 5:**
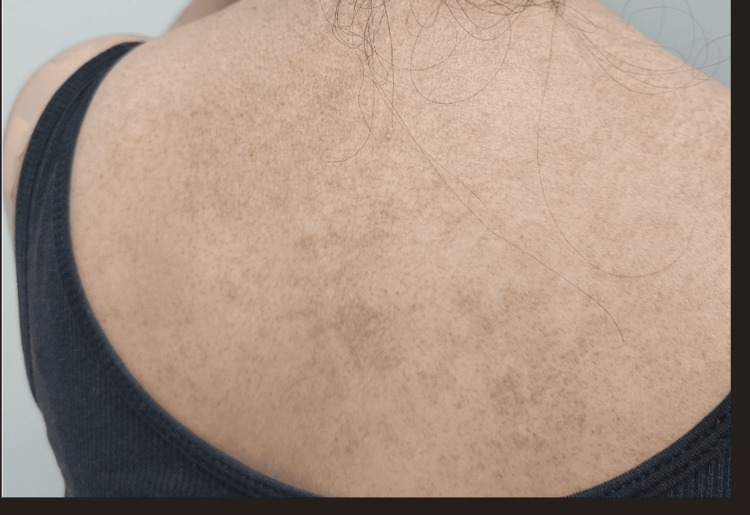
Painful pruritic rash on the upper back improved after anifrolumab treatment

A 51-year-old Hispanic female, with no past medical history, was hospitalized for weakness in the hip flexors and shoulder abductors. Her creatine phosphokinase (CPK) peaked at 7002 IU/L (normal: 2-200 IU/L). Intravenous methylprednisolone was administered. She had a violet rash on her eyelids (Figure 1), post-inflammatory pigmentation on the face, and a dusky pruritic rash on her scalp, chest (Figure 2), and upper back (Figure 3). Gottron's papules (Figure 4), inverse Gottron's sign, and an abnormal nailfold capillary exam were observed. DM was diagnosed based on the clinical manifestations of proximal muscle weakness and elevated CPK. Lab studies revealed a positive anti-Mi-2 antibody and anti-nuclear antibody (ANA) (1:320 homogeneous pattern). Other tests, including rheumatoid factor, anti-cyclic citrullinated peptide, extranuclear antibody panel (ENA), and COVID-19 antibodies, were negative. Her erythrocyte sedimentation rate was 40 mm/hr, and her C-reactive protein was normal. A computed tomography (CT) scan showed pulmonary nodules but no malignancy. She was started on 60 mg prednisone taper, HCQ 400 mg daily, MTX 20 mg weekly, and folic acid (FA) daily. Due to persistent muscle weakness, intravenous immunoglobulin (IVIG) 2 g/kg was added. She initially had improvement in her muscle strength, and CPK normalized to 263 IU/L. She was tapered off prednisone and continued IVIG, MTX, HCQ, and weekly FA. Despite initial improvement, her dermatitis relapsed with a painful and pruritic rash on the scalp, face, chest, back, abdomen, and extremities. Biopsies showed interface dermatitis, indicating a DM recurrence. Transitioning to MMF did not improve the rash, and high-dose prednisone was needed. A magnetic resonance imaging (MRI) of the left shoulder showed inflammatory changes suggestive of coexistent rheumatoid arthritis (RA). Additional testing revealed positive anti-carbamylated peptide antibody (anti-CarP). Subsequently, tofacitinib for five months followed by rituximab failed to manage the rash, and methylprednisolone tapering was unsuccessful. Tumor necrosis factor inhibitors were avoided due to the risk of systemic lupus erythematosus (SLE)-like drug eruption. A repeat CT scan did not show signs of malignancies. Anifrolumab was initiated monthly, which significantly improved the rash and pruritis just after two infusions (Figure 5 shows improvement in the rash on her upper back). She was successfully tapered off prednisone to 5 mg daily.

## Discussion

Based on our research, this is the second case report that has reported success in treating refractory cutaneous symptoms of DM with anifrolumab after failing standard and multiple therapeutic combinations [6]. Anifrolumab is a type 1 interferon receptor inhibitor (IFN-1i) human monoclonal antibody used to treat SLE [7]. It is FDA-approved for non-renal manifestations of SLE. This IFN pathway seems to be active in patients with DM [8] as the presence of IFN-1 and IFN-2 has been reported in muscle biopsies of patients with inflammatory myopathies. Moreover, the IFN activation signature is present in the muscle, blood, and skin of patients with DM [9]. IFN-1 activates toll-like receptors which activate the dendritic cells leading to the secretion of cytokines and chemokines [9]. This potential pathophysiological role of IFN in DM may explain the cutaneous improvement in our patient with anifrolumab. Anifrolumab has a good safety profile in SLE patients with three-year data on background conventional disease-modifying antirheumatic drug (DMARD) therapies [10].

## Conclusions

SLE and DM share similarities in their pathophysiology and cutaneous disease involvement. They can be differentiated clinically. We aim to highlight the benefits of anifrolumab for treating DM refractory skin manifestations.
